# The method of Katharina Schroth - history, principles and current development

**DOI:** 10.1186/1748-7161-6-17

**Published:** 2011-08-30

**Authors:** Hans-Rudolf Weiss

**Affiliations:** 1 Orthopedic Surgeon, Physical Medicine and Rehabilitation, Chiropractor (German school) Rehabilitation service Gesundheitsforum Nahetal Alzeyer Str. 23 D-55457 Gensingen, Germany

## 

Katharina Schroth, born February 22nd 1894 in Dresden Germany, was suffering from a moderate scoliosis herself and underwent treatment with a steel brace at the age of 16 years before she decided to develop a more functional approach of treatment for herself.

Inspired by a balloon, she tried to correct by breathing away the deformities of her own trunk by inflating the concavities of her body selectively in front of a mirror. She also tried to ‚mirror' the deformity, by overcorrecting with the help of certain pattern specific corrective movements. She recognized that postural control can only be achieved by changing postural perception.

From 1921 this new form of treatment with specific postural correction, correction of breathing patterns and correction of postural perception was performed with rehabilitation times of 3 months in her own little institute in Meissen and in the late 30's and early 40's she was supported by her daughter, Christa Schroth.

After World War II, Katharina Schroth and her daughter moved to West Germany to open a new little institute in Sobernheim, which constantly grew to a clinic with more than 150 in-patients at a time, treated as a rule for 6 weeks. In the 80's this institute was renamed to ‚Katharina Schroth Klinik'. At this time the first studies were carried out and the patient series for the first prospective controlled trial was derived from the patient samples of 1989-1991.

Content, rehabilitation times and patients meanwhile have changed, and braces have been developed to offer highest treatment security.

Therefore today, bracing in the patient at risk has to be regarded as the primary treatment. We have been able to reduce the training times by adapting the old techniques and introducing new forms of postural education (sagittal correction, ADL correction and experiential learning) whilst the programme is still based on the original approaches of the 3-dimensional treatment according to Katharina Schroth, namely specific postural correction, correction of breathing patterns and correction of postural perception.

### History of Katharina Schroth's method of scoliosis treatment

The history of conservative treatment of scoliosis is rather long and leads us back to the original methods of Hippocrates (460-370 BC) [1]. Although more than two thousand years have passed since the century of Hippocrates, the main approach of conservative scoliosis treatment has been based on mechanical viewpoints still in the early 20th Century and in most of the approaches still existing today. Correction exercises were widely distributed in whole of Europe during the last two centuries; some of them were using three therapists for one patient (Figure 1) during scoliosis correction [2].

**Figure 1:**
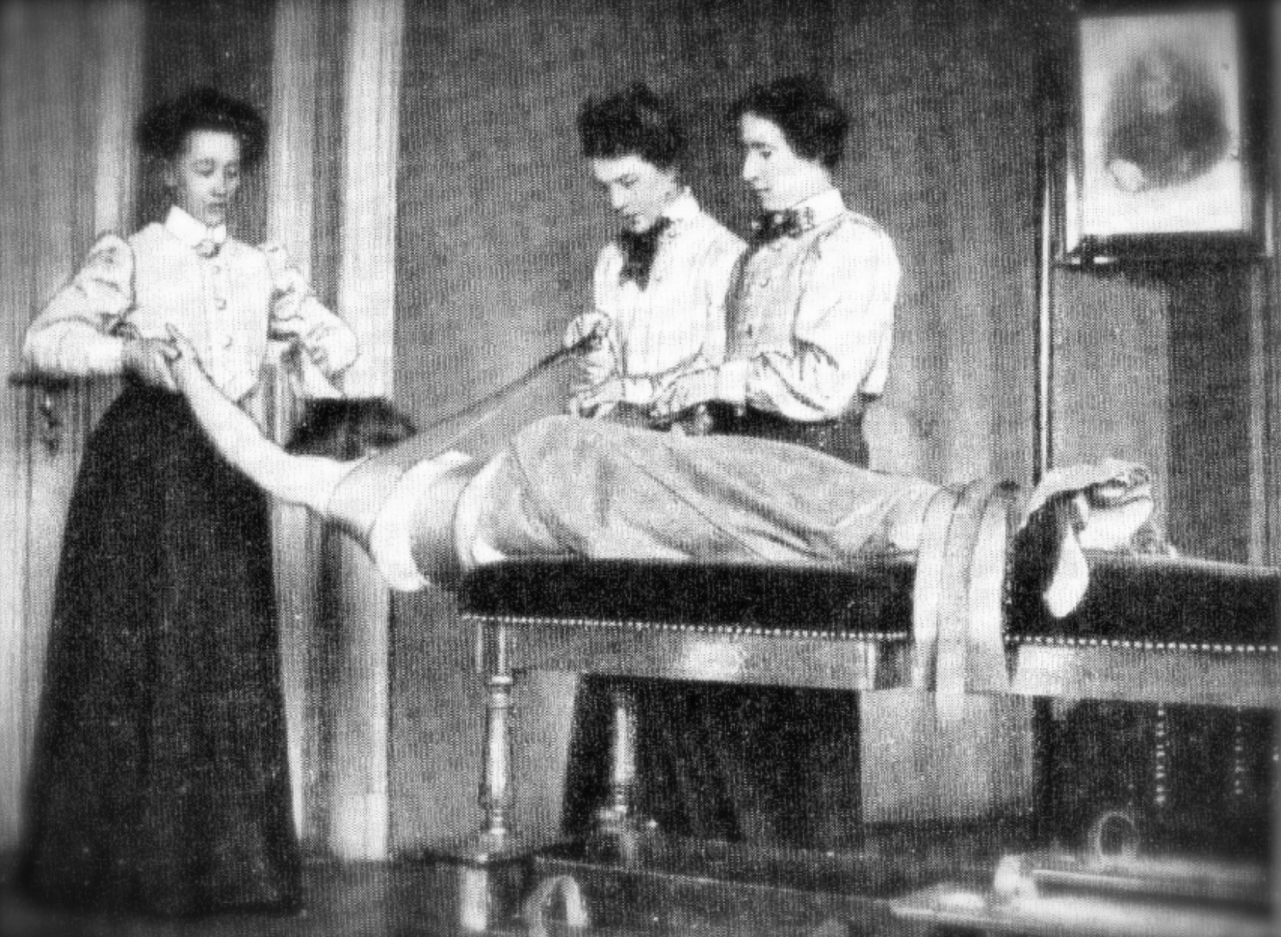
**Mechanical approach with curvature redression with the help of three therapists in the approach used by Oldevig **[2,12].

The history of the Schroth method is a history involving the professional work of three generations. The initiation of the programme was the result of Katharina Schroths studies (Additional file 1 and 2), in part a development from studying her own body, her own spinal function and the corrective movements possible. Mirror monitoring plays an important role in the original Schroth programme so as to allow synchronizing the corrective movement and the postural perception with the visual input (Figure 2). As breathing and its functional correction played an important role, her first pamphlet focused on breathing in general [3] and later on also describing the importance of postural perception by the patient and its improvement with the help of specific correction exercises [4,5].

**Figure 2:**
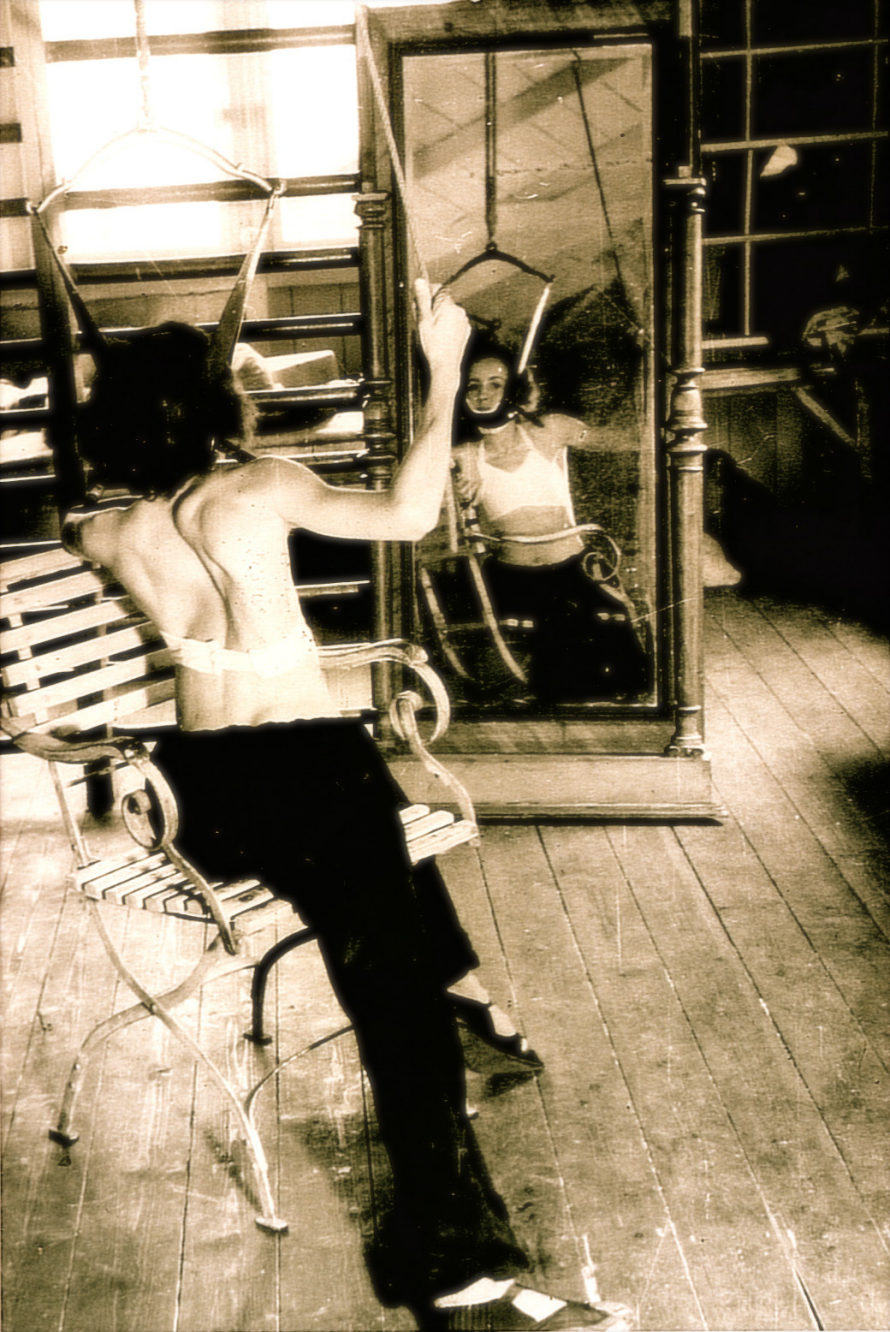
**Patient with a large thoracic curvature exercising on her own in front of a mirror**. Mirror monitoring plays an important role in the original Schroth programme so as to allow synchronizing the corrective movement and the postural perception with the visual input [12]. [Historical picture from the picture database of Christa Lehnert-Schroth, Meissen 1944].

In the 70's Christa Lehnert-Schroth further developed the method and introduced a simple classification, which is still used today by physiotherapists (Figure 3). Additionally, she discovered the importance of the lumbosacral (counter-) curve (4th Curve) for pattern specific postural correction and described all this in her book, which was first published in 1973 and is now available in the 7th edition [6]. This historically important book is also available in English and Korean [7].

**Figure 3:**
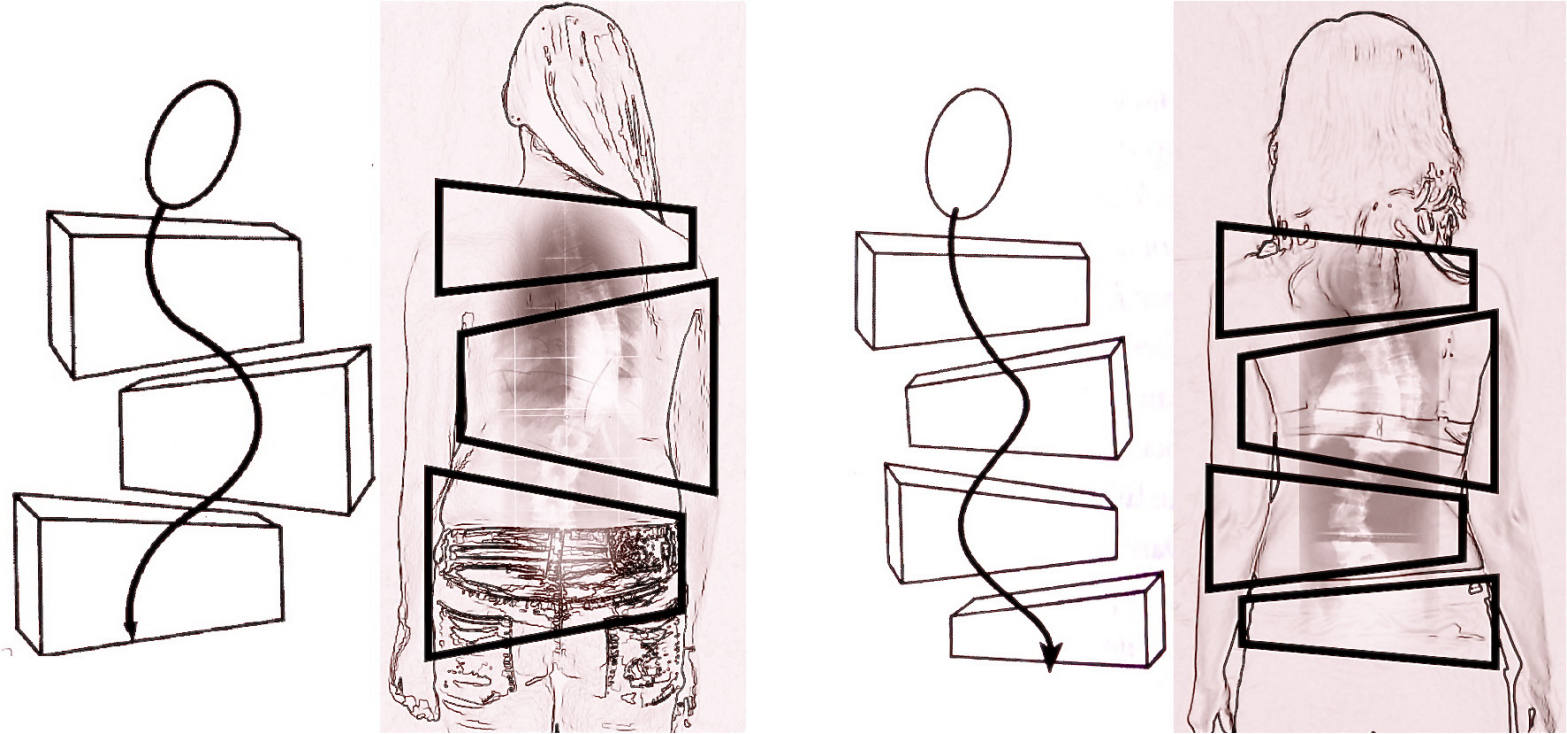
**The original classification according to Lehnert-Schroth**. On the left the *Three Curve Pattern *with the shoulder, thoracic and lumbo-pelvic block deviated against each other in the frontal plane and also rotated against each other. On the right the *Four Curve Pattern *with a separation of the lumbo-pelvic block into a lumbar and a pelvic block deviated against each other in frontal plane and also rotated against each other. Per definition: the pelvic block symbolises the lumbosacral counter curve and this curve is defined as the 4th Curve [12].

In the 90's, Dr. Rigo and the author constantly improved the programme and as a result of this collaboration the book ‚Befundgerechte Physiotherapie bei Skoliose' was written by both of them (1st edition 2001) until the second edition appeared in 2006 [8] and the book was translated into Spanish [9].

In 2010 the latest developments were published including new educational approaches and the correction of the sagittal profile [10,11] and now the 3rd edition of the German book ‚Befundgerechte Physiotherapie bei Skoliose' is dedicated to these new aspects [12].

The history of all this, however began in East Germany in the first decade of the last century:

#### How it all started

Katharina Schroth, born February 22nd 1894 in Dresden Germany, was suffering from a moderate scoliosis herself and underwent treatment with a steel brace at the age of 16 before she decided to develop a more functional approach of treatment for herself (1910).

Inspired by a balloon, she tried to correct by breathing away the deformities of her own trunk by inflating the concavities of her body selectively in front of a mirror. She also tried to ‚mirror' the deformity, by overcorrecting with the help of certain pattern specific corrective movements. Additionally, she recognized that postural control can only be achieved by changing postural perception. These aspects were published as early as 1924 and later on [3-5] and were elaborated even more during the first decade of her professional career as a gymnast.

Katharina Schroth began her professional life as a teacher at a Business & Language school, however she decided to leave this field and undergo training at a gymnast's school in order to be able to treat patients herself.

From 1921 on this new form of treatment with specific postural correction, correction of breathing and correction of postural perception was performed with rehabilitation times of three to sometime six months in her own little institute in Meissen (Figure 4, 5) and from the late 30's she was supported by her daughter, Christa Schroth (Figure 6, 7).

**Figure 4:**
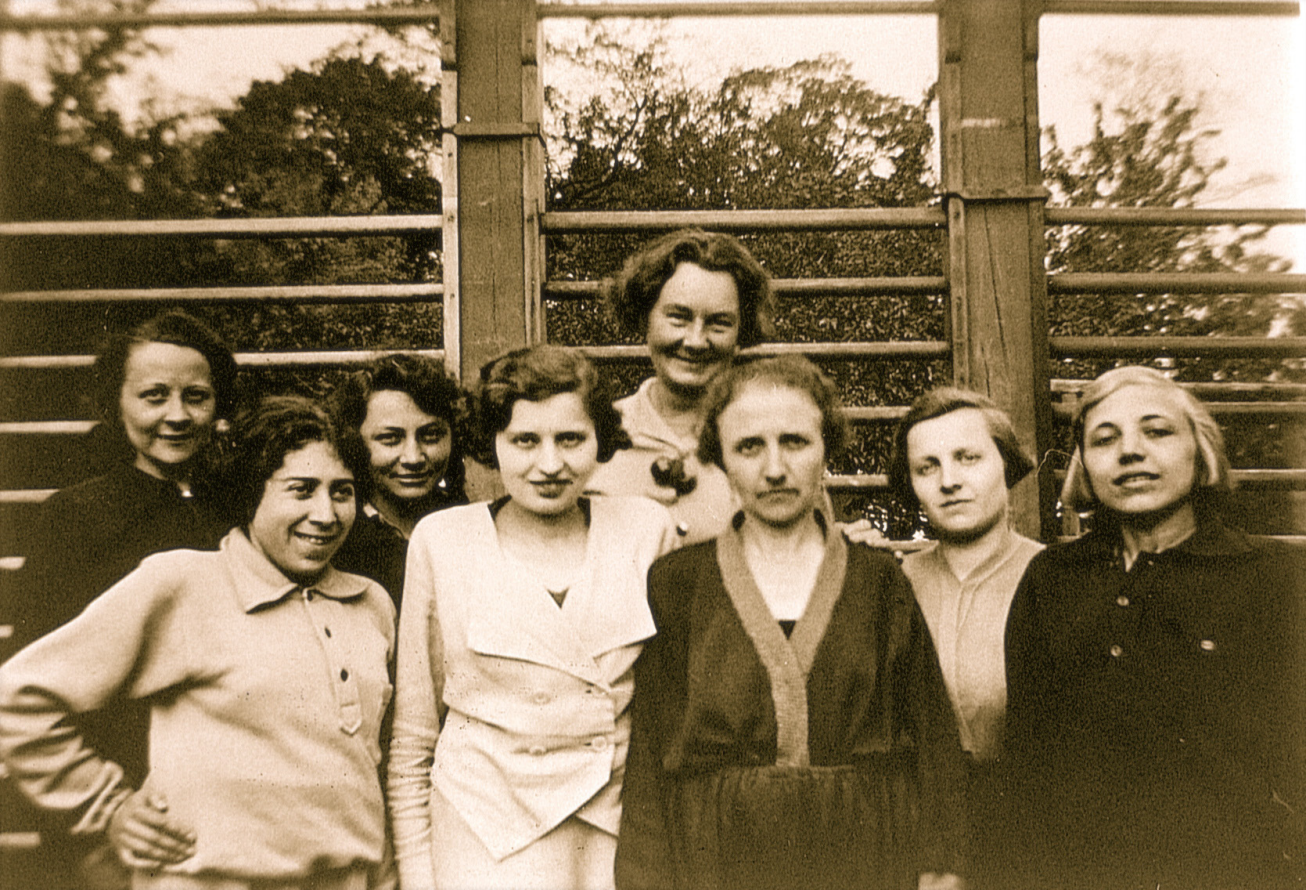
**Katharina Schroth (centre in the background) seen with her patients in the 30's**. [Historical picture from the picture database of Christa Lehnert-Schroth].

**Figure 5:**
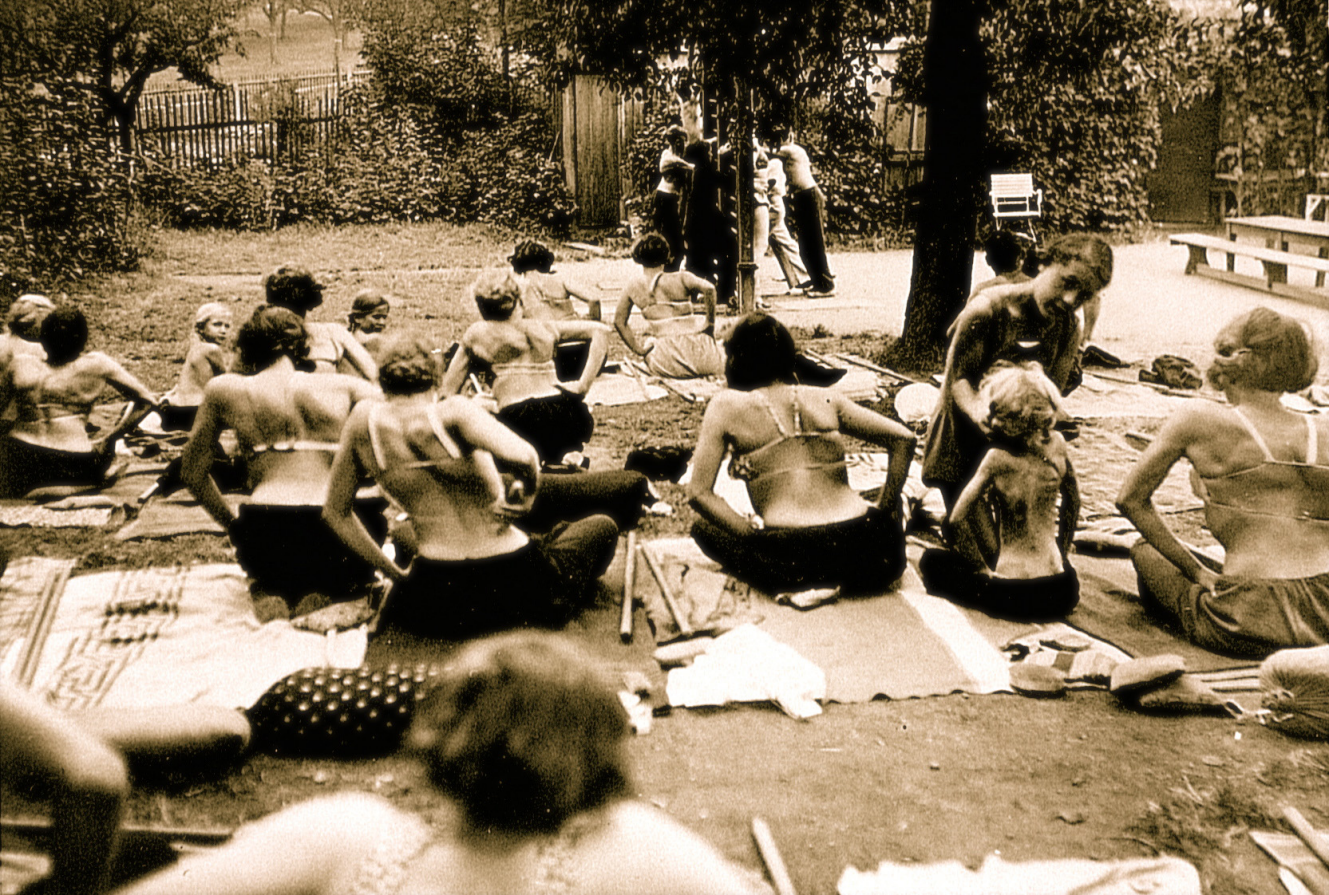
**A group of patients with large curvatures exercising in the garden of the little institute run by Katharina Schroth in the 30's in Meissen**. [Historical picture from the picture database of Christa Lehnert-Schroth].

**Figure 6:**
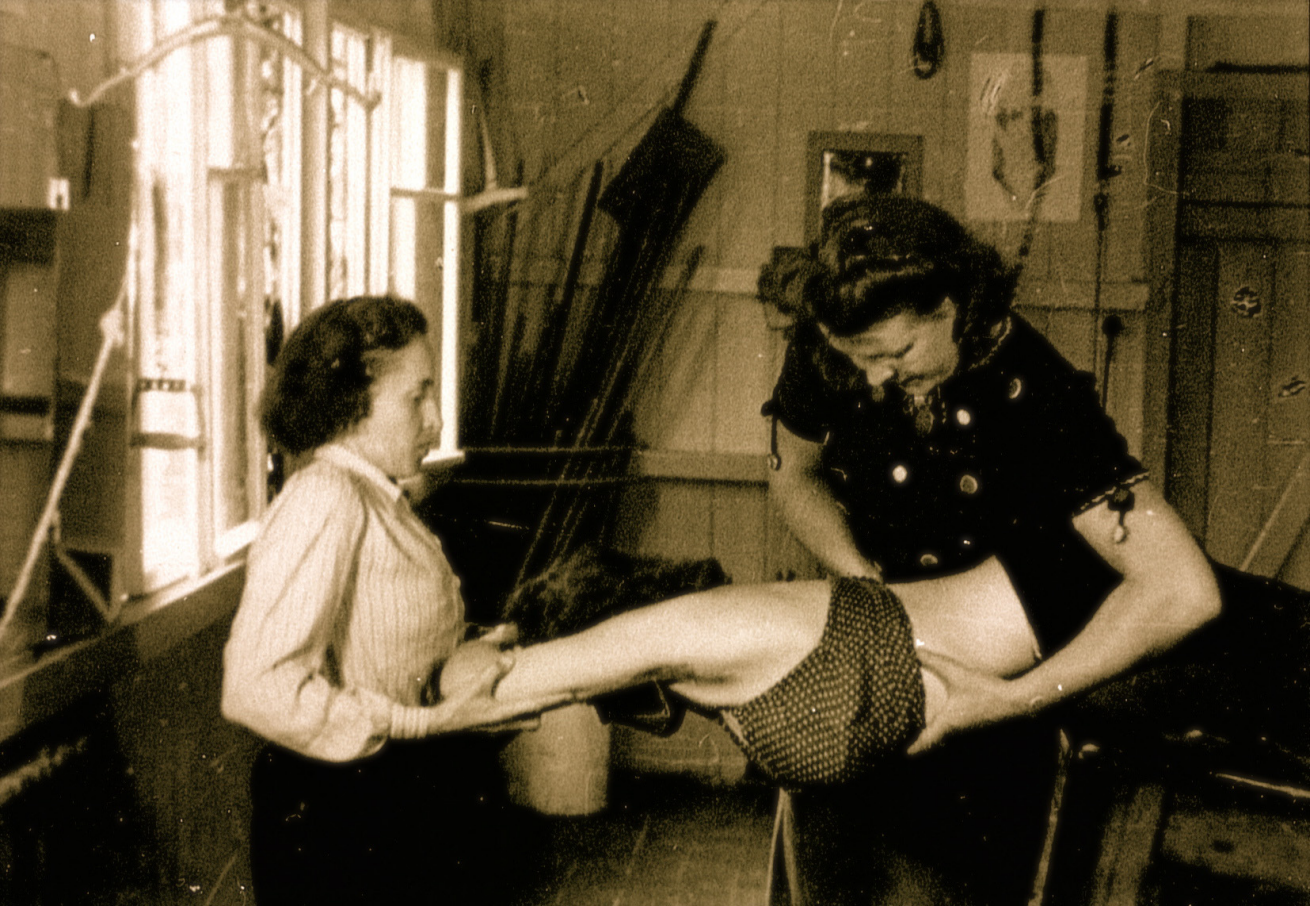
**Individual training of a patient by Christa Schroth, daughter of Katharina Schroth in the 40's**. [Historical picture from the picture database of Christa Lehnert-Schroth].

**Figure 7:**
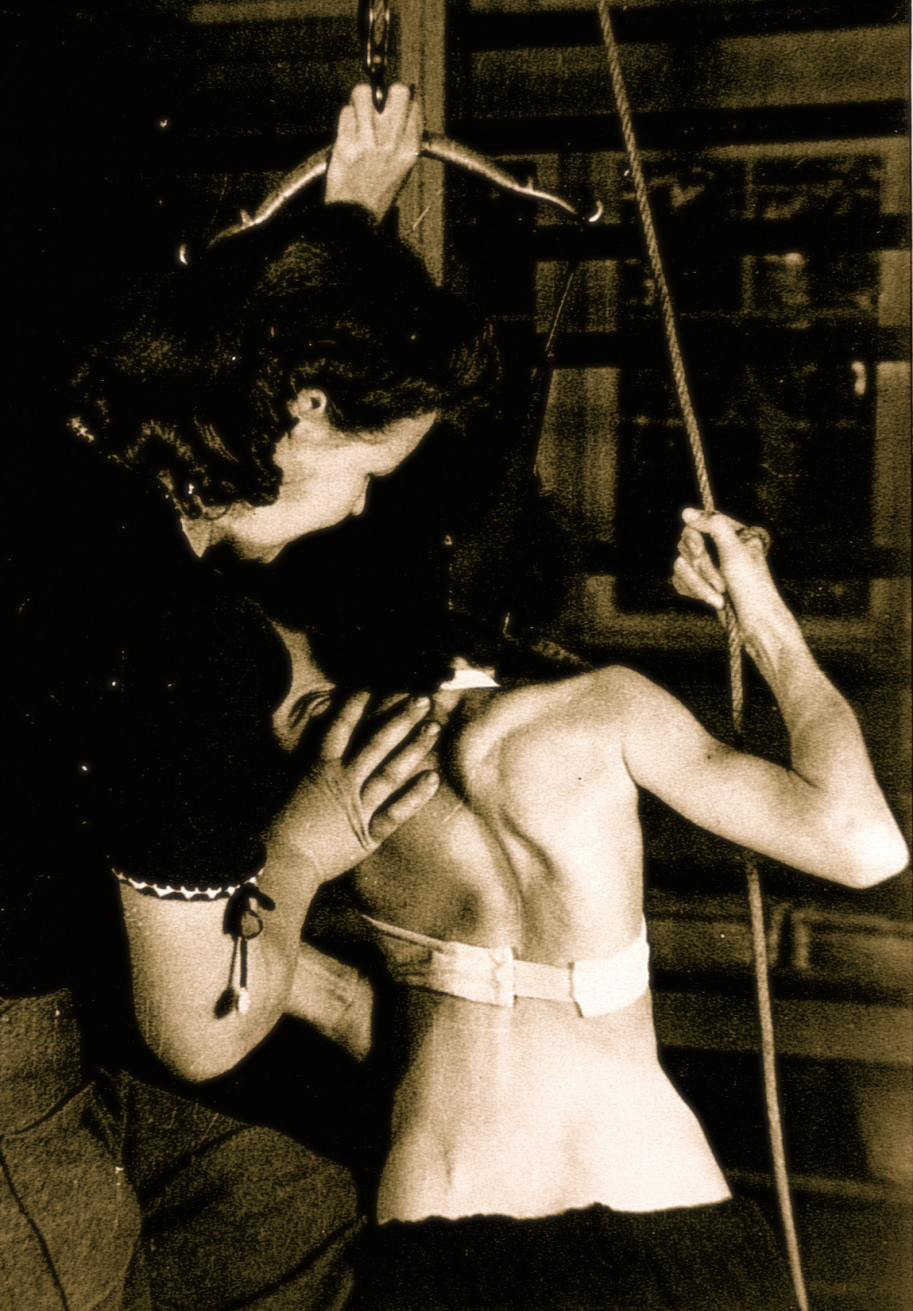
**Individual training of another patient by Christa Schroth, daughter of Katharina Schroth in the 40's**. [Historical picture from the picture database of Christa Lehnert-Schroth].

During that time, patients with curvatures exceeding 80° with huge rib humps and very stiff deformities of different origins were the main attraction (Figure 8, 9 and 10).

**Figure 8:**
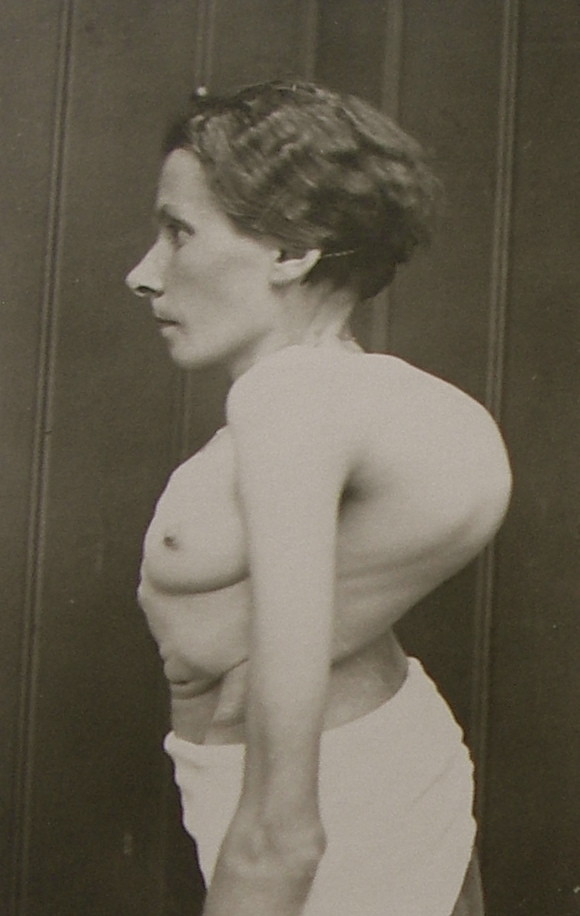
**A typical patient with a large curvature as treated in Katharina Schroth's first institute in the 30's in Meissen**. [Historical picture from the picture database of Christa Lehnert-Schroth].

**Figure 9:**
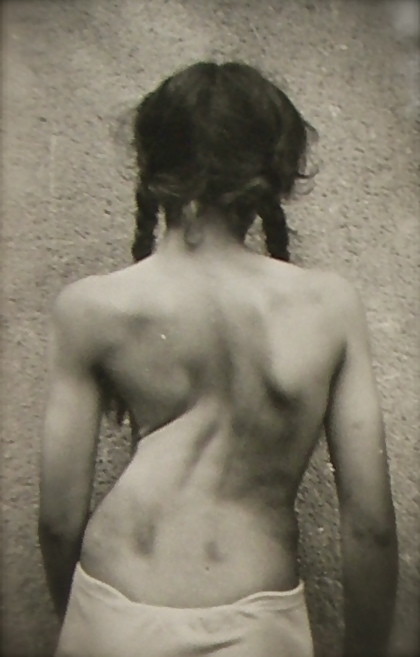
**Another typical patient with a large curvature as treated in Katharina Schroth's institute**. [Historical picture from the picture database of Christa Lehnert-Schroth, Gottleuba 1950, second Schroth institute, East Germany].

**Figure 10:**
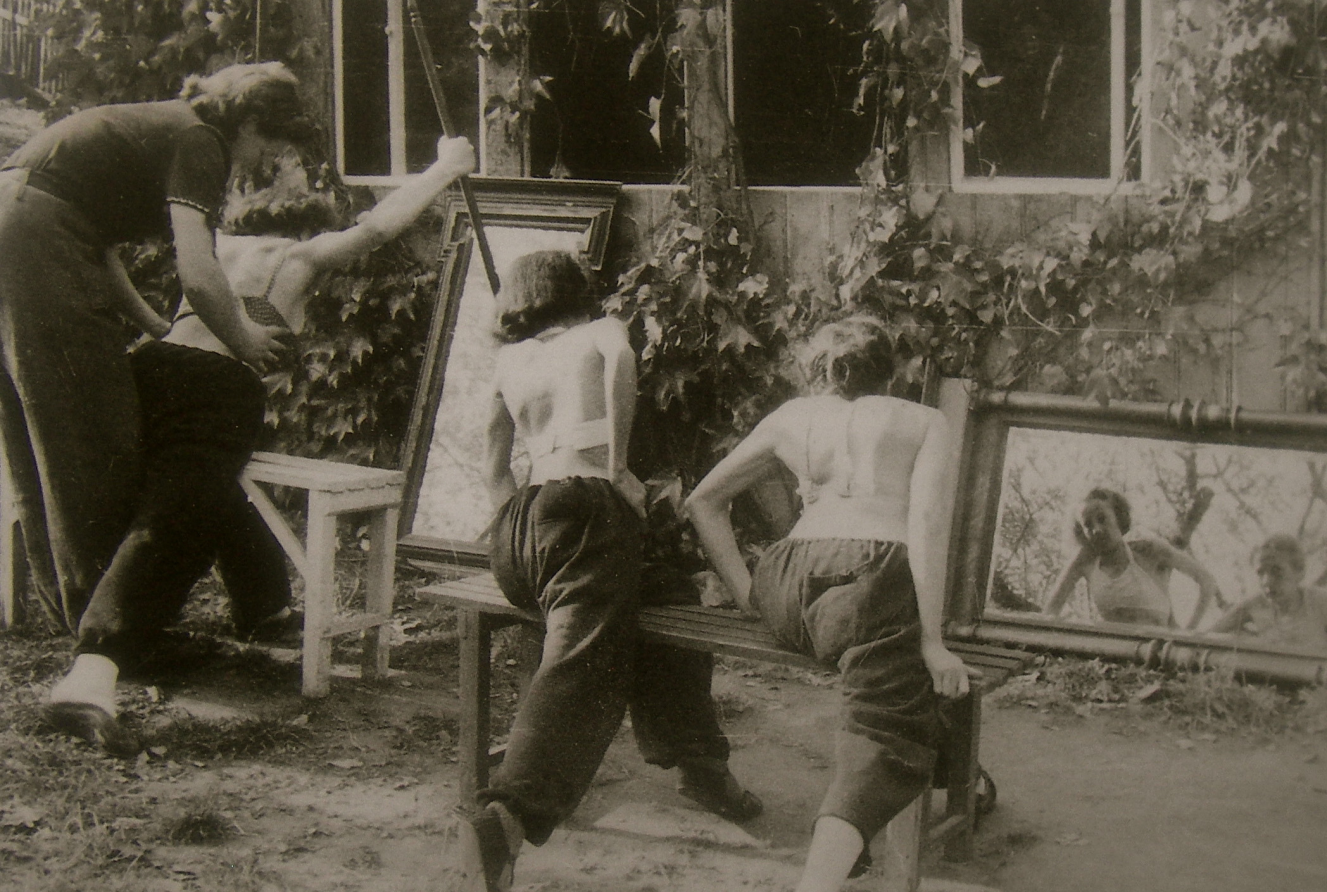
**A small group of patients with large curvatures exercising in front of mirrors to allow the monitoring of the progress of correction**. [Historical picture from the picture database of Christa Lehnert-Schroth, Meissen 1944].

Besides individual exercises, also with passive manual correction by a therapist, a group setting was established allowing the treatment of patients with similar curve patterns in one group (Figure 11).

**Figure 11:**
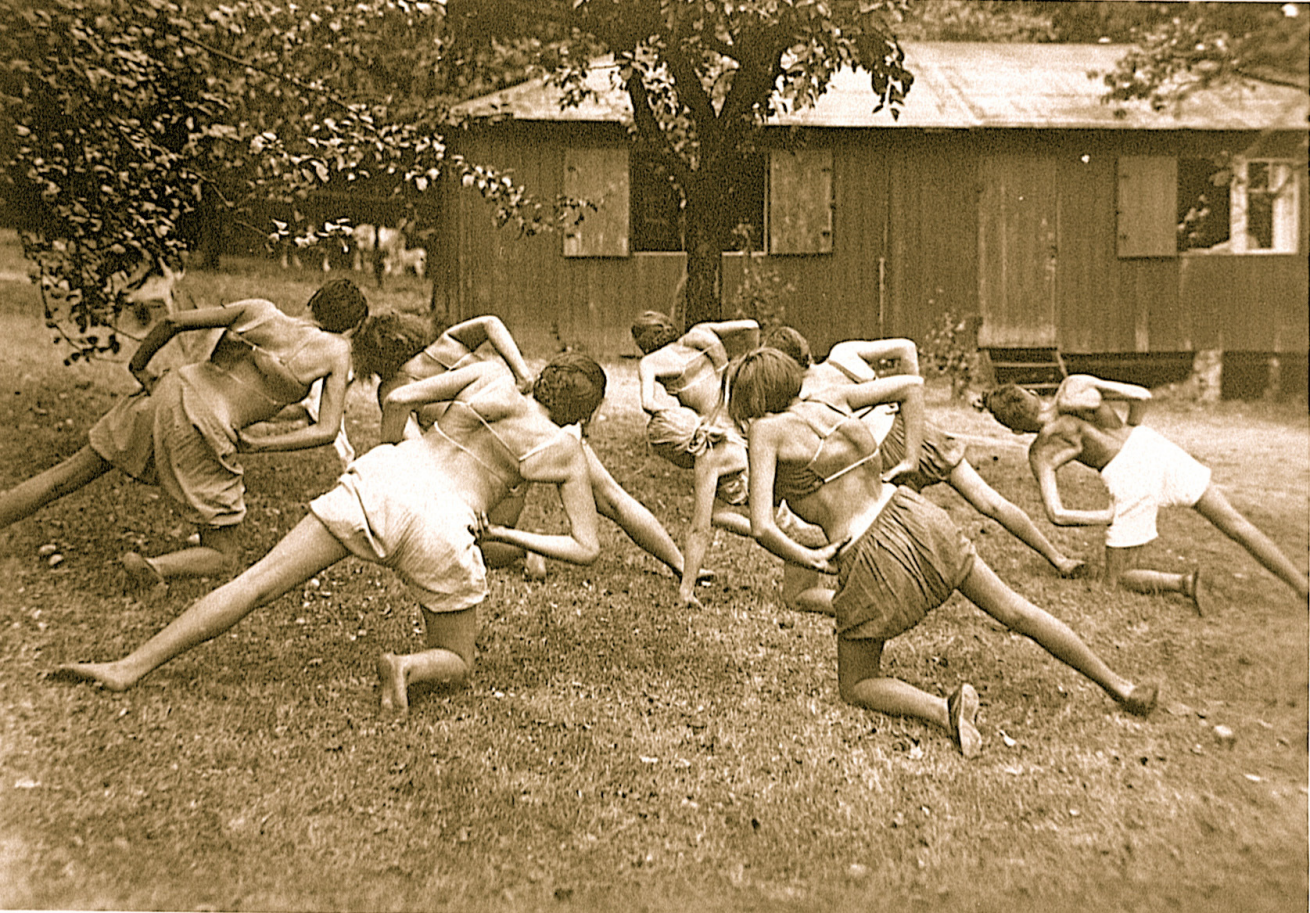
**A group of patients with major thoracic curvatures exercising the ‚muscle cylinder'**. [Historical picture from the picture database of Christa Lehnert-Schroth, Meissen in the 30's].

The institute had a large garden and a little hut with some helpful tools for individal and group treatment. Most of the treatment was carried out in the garden, fresh air and sunrays increased the patient's general health at a time where people were not used to exposing their skin to the sun or indeed to other people (Figure 12).

**Figure 12:**
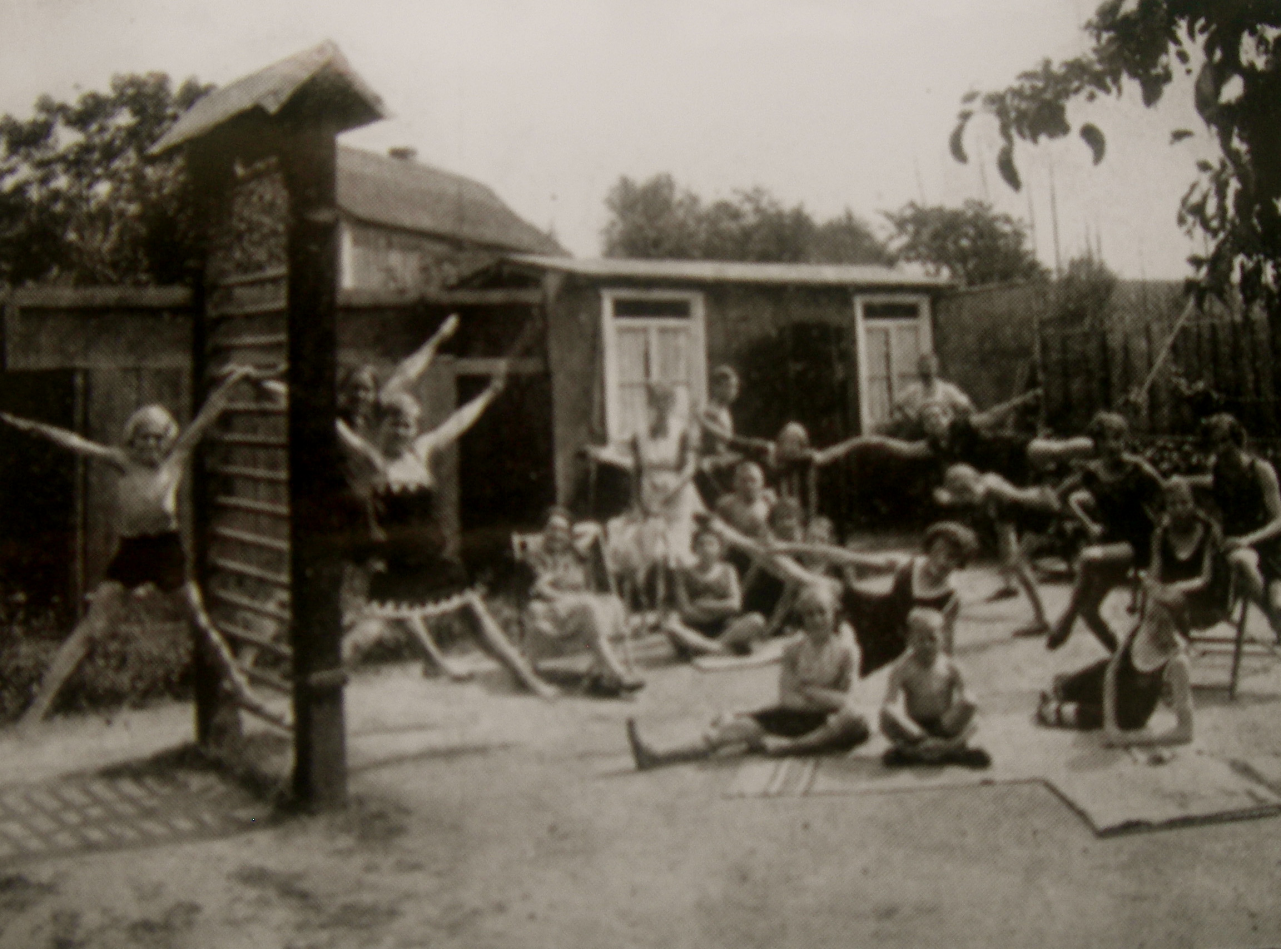
**Patients in front of the little hut of the institute**. The patients were used to exercise in the garden and only when there was rain the treatment took place in the hut [Historical picture from the picture database of Christa Lehnert-Schroth, Meissen 1935].

Mirror monitoring has always been important as can be seen in Figure 2 and 10 in an individual session of patients in front of a mirror treated by Christa Schroth in the 40's.

Franz Schroth, Katharina Schroth's husband, also helped in the first institute with individual corrections and special strengthening exercises (Figure 13).

**Figure 13:**
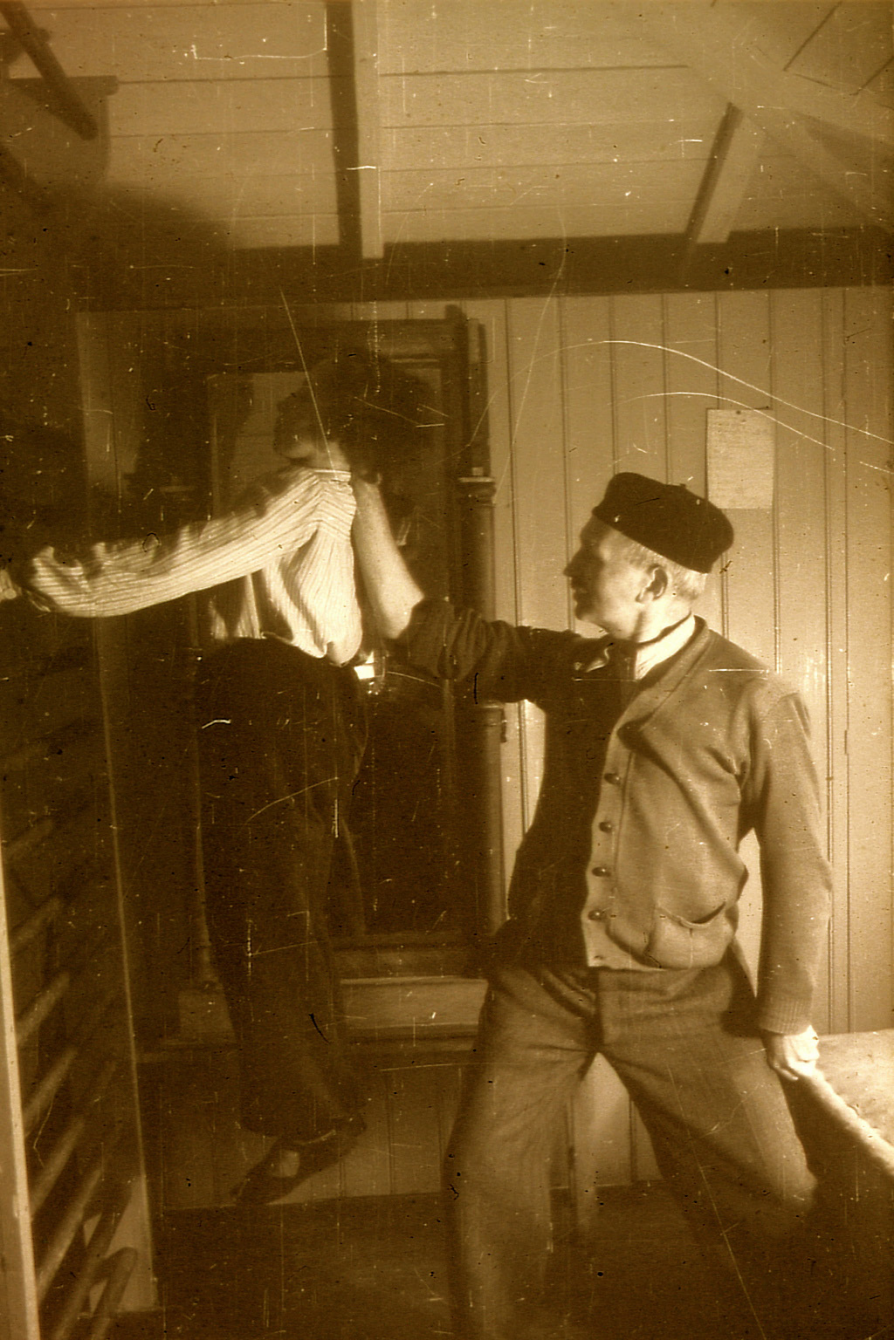
**Franz Schroth, Katharina Schroth's husband, assisted in patient training regularly**. [Historical picture from the picture database of Christa Lehnert-Schroth].

As early as in the late 20's of the last century a battle of methods began. A Professor from Leipzig (Prof. Scheede), where Hoffa exercises were performed, fought against the little centre of Katharina Schroth heavily as she was neither a professional trainer, nor a physician, but had started her programme as a schoolteacher who followed a class of gymnasts after she had started her insitute.

After World War II Katharina Schroth was forced to leave her little institute in Meissen. Before she went to the West she was employed by the state to offer her services together with her daughter in a medical centre at Gottleuba during the early 50's.

#### New start in the West

After World War II, Katharina Schroth and her daughter moved to West Germany to open a new little institute in Sobernheim in the early 60's, which constantly grew to a clinic with sometimes more than 150 in-patients treated as a rule for 6 weeks (Figure 14 and 15). After her divorce from her first husband, Ernst Weiss, Christa Schroth married Adalbert Lehnert, who helped her to build up this new centre and who was also involved in the treatment of patients (Figure 16).

**Figure 14:**
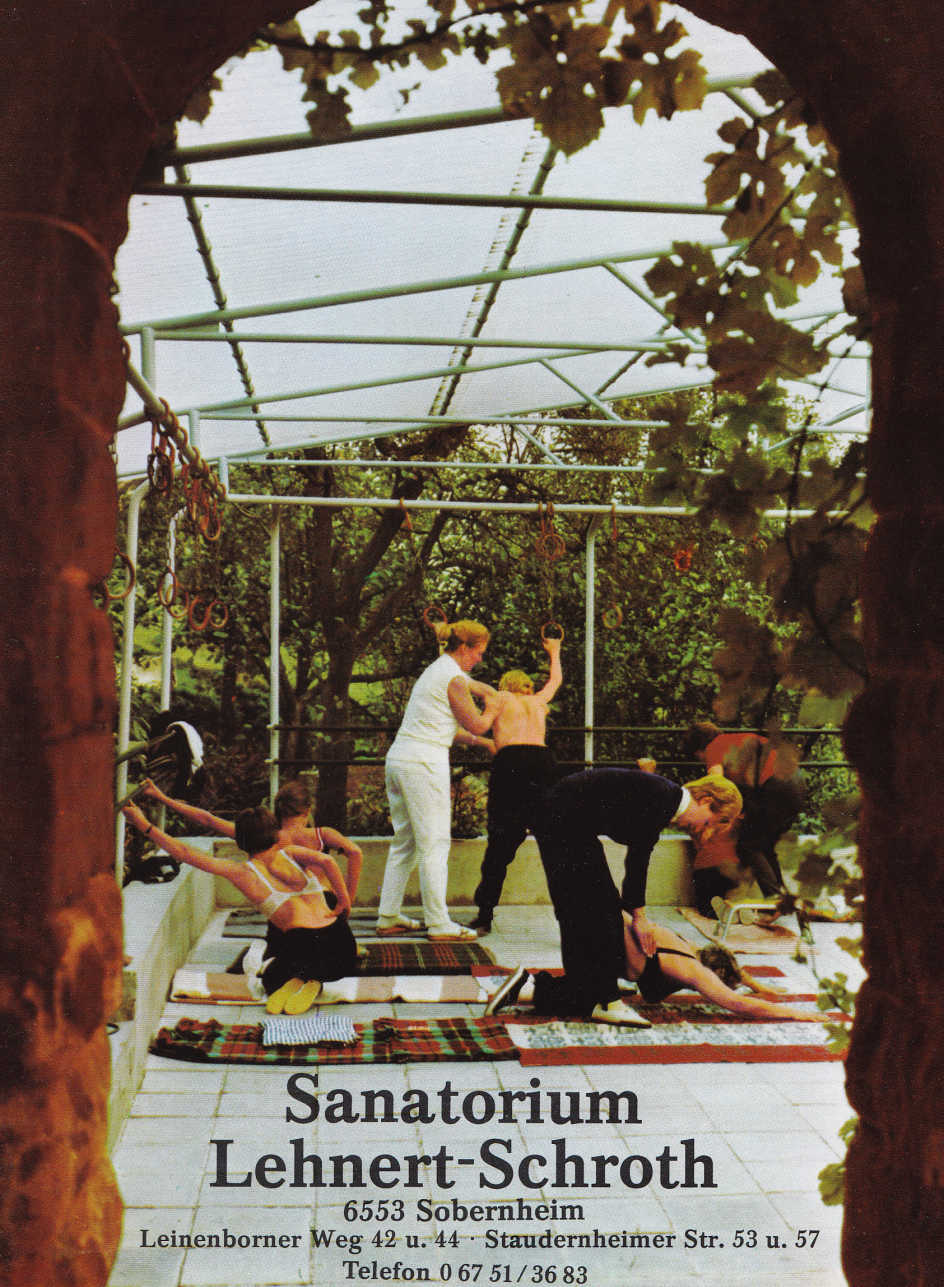
**Christa Lehnert-Schroth, Katharina Schroth's daughter, amidst a group of patients in her new institute in Sobernheim (Folder of the ‚Sanatorium Lehnert-Schroth in the 70's)**. [Historical picture from the picture database of Christa Lehnert-Schroth].

**Figure 15:**
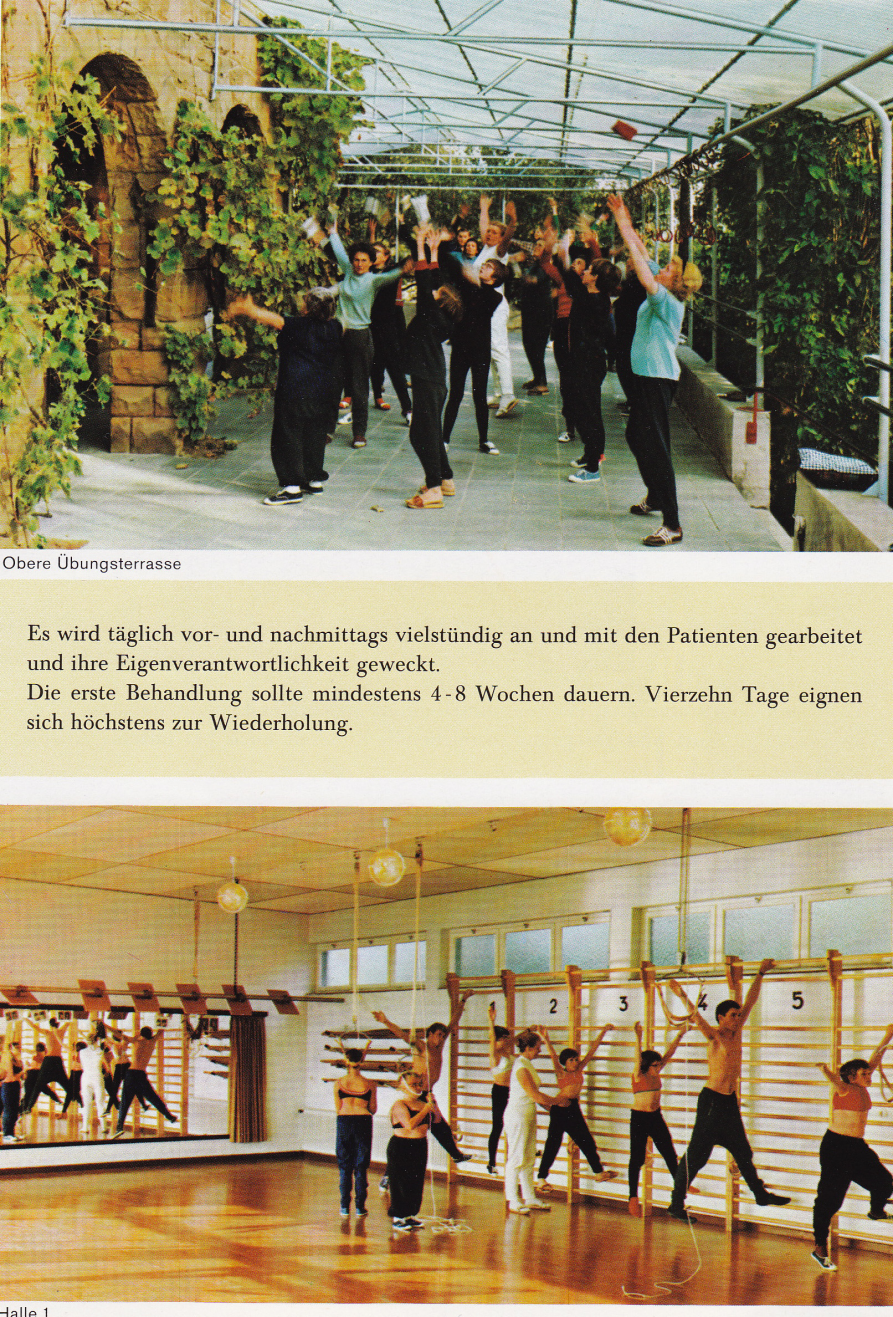
**Exercise setting in the new institute in Sobernheim (Folder of the ‚Sanatorium Lehnert-Schroth in the 70's)**. [Historical picture from the picture database of Christa Lehnert-Schroth].

**Figure 16:**
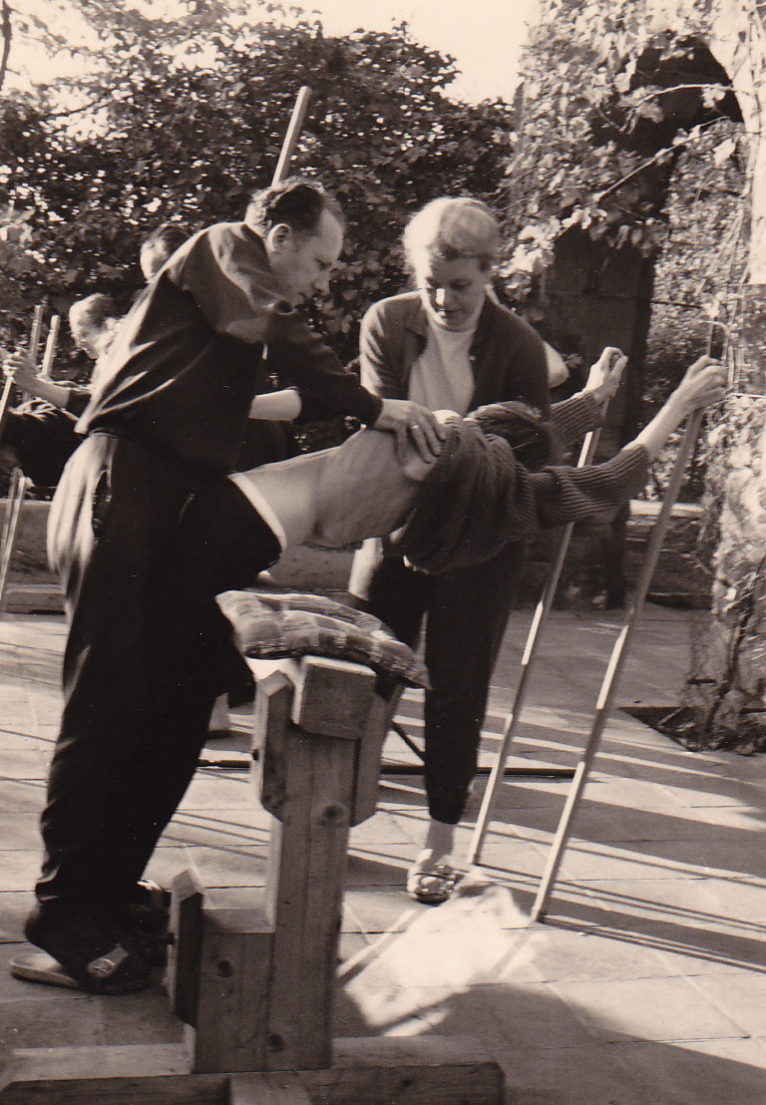
**Adalbert Lehnert and Christa Lehnert-Schroth treating a patient with significant rib hump together in the early 70's in the new institute in Sobernheim**. [Historical picture from the picture database of Christa Lehnert-Schroth].

In the 70's a series of investigations were carried out with respect to vital capacity improvements and improvement of cardiopulmonary function contributing to the acknowledgement of the method at some universities [13,14].

It was also in the 70's, when the impact of the lumbosacral curve on the correction of certain curve patterns was discovered [15,16].

Christa Lehnert-Schroth recognized the spontaneous correction of a functional leg length discrepancy just by straightening the lumbar curve [15].

In the 80's the institute, ‚Sanatorium Lehnert-Schroth' was renamed to ‚Katharina Schroth Klinik' while Katharina Schroth was not as active as in the 60's and early 70's. Nevertheless, she fought constantly for her method of treatment and had lots of arguments with professors from different German universities.

More emphasis at that time was laid upon the correction of pelvic asymmetries to address the lumbosacral curve and unfortunately the powerful corrections initially defining the treatment of Katharina Schroth were increasingly lost.

This was the time of making the treatment more and more complicated, focusing on little deviations while the main curvature correction was drifting out of sight.

More patients with curvature angles of less than 40° and typical flatback deformities were treated, but there was no real development towards a systematical correction of the sagittal profile. While the original programme was for thoracic curves exceeding 80° with trunk rotations and rib humps leading to a more kyphotic inclination of the trunk, the moderate curvatures were addressed quite well in the frontal and coronal plane, but the sagittal profile was still underestimated. The only correction of a thoracic flatback was through rotational breathing while the starting positions of the exercises was still with both arms in elevation increasing the flatback deformity (Figure 17 and 18).

**Figure 17:**
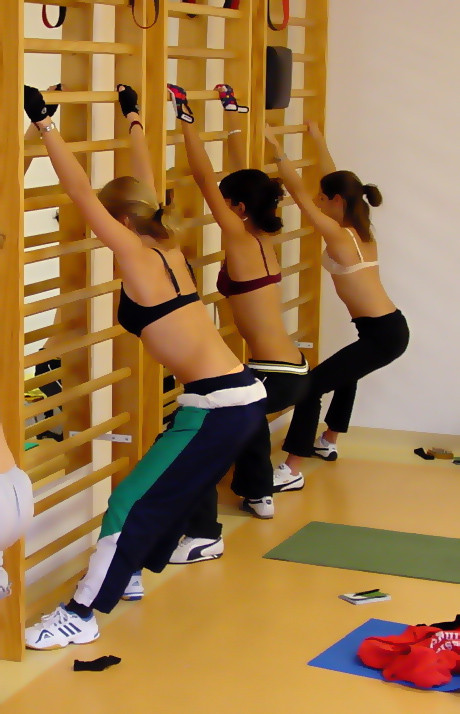
**Typical exercise setting in the Katharina Schroth Klinik in Bad Sobernheim**. The elevation of both arms leads to an increase of the flatback deformity [12].

**Figure 18:**
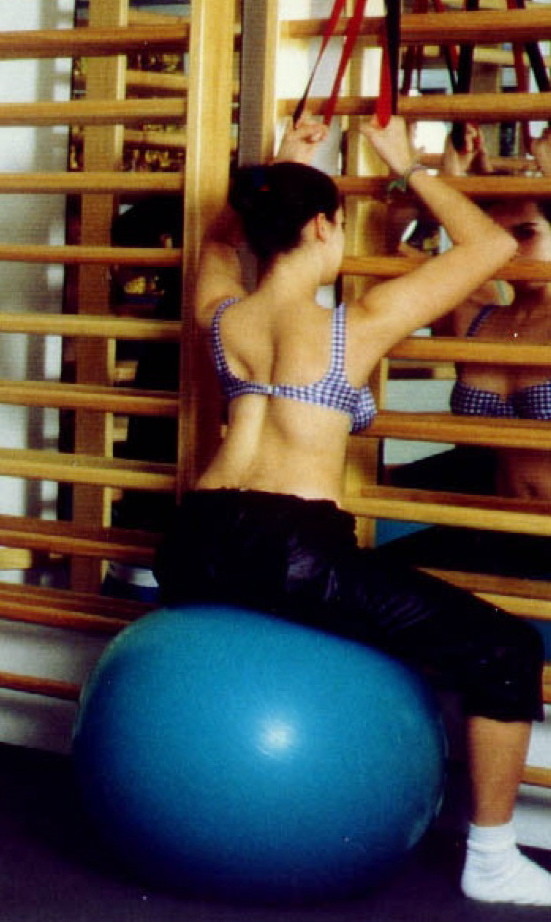
**Another patient in the typical exercise setting in the Katharina Schroth Klinik in Bad Sobernheim**. The elevation of both arms leads to an increase of the flatback deformity [12].

#### First investigations-first scientific evidence

At this time, first studies were completed and the patient series for the first prospective controlled trial was derived from the patient samples of 1989-1991, a sample first published in 1995 as a prospective study in German [17], 1997 in English [18] and later on including age and sex matched controls from another regional study on untreated patients as a prospective controlled study [19]. Studies on the improvement of cardiopulmonary capacity, vital capacity improvement, electromyography and influence of the treatment of pain followed [20-24].

Most of the studies were cohort studies in a pre-/postintervention design and there were no mid- or long-term follow-ups. Nevertheless, huge numbers of patients were investigated. 794 Patients were investigated with the ECG showing that even signs of manifest right cardiac strain were reduced highly significantly after an in-patient rehabilitation of 6 weeks using the Schroth programme [20]. More than 800 Patients were material for the study on vital capacity and rib mobility published in Spine 1991 [21], the material in the study on muscle activity reductions after intensive rehabilitation consisted of more than 300 patients [22].

The only mid-term study with a follow-up of more than a 30 months period was the one with the cohort treated between 1989 and 1991 first published in the English language in 1997 [18], which was the basis for our prospective controlled trial published in 2003 [19].

During the 90's there was some development with respect to the correction of thoracolumbar curves including the derotational effect of the psoas muscle. More and more exercises were performed in horizontal positions with as many corrective tools as possible, surely not available during the patients' home programmes (Figure 19).

**Figure 19:**
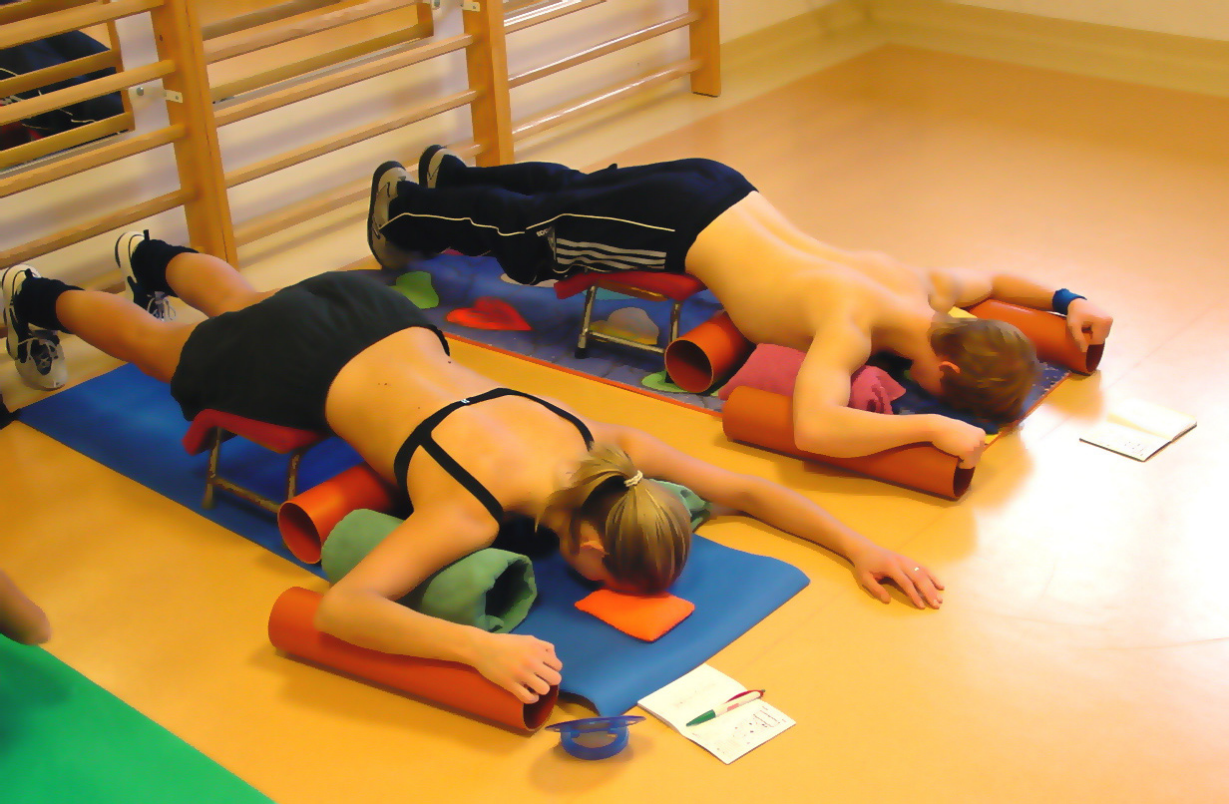
**Typical treatment in the Asklepios centre in Bad Sobernheim with very many tools not available at home, lying on the floor not using the automated postural correction by using the corrective postural reflex activation **[12].

In the 80's the author performed an analysis of the different aspects of the original Schroth method [25]. One of the most important factors of the original Schroth method was the automated precorrection of the deformity with the help of postural reflex activity in certain asymmetric upright starting positions. The exercise began precorrected with the help of postural reflex activity in upright asymmetric starting positions and the exercise itself increased this precorrection (Figure 20).

**Figure 20:**
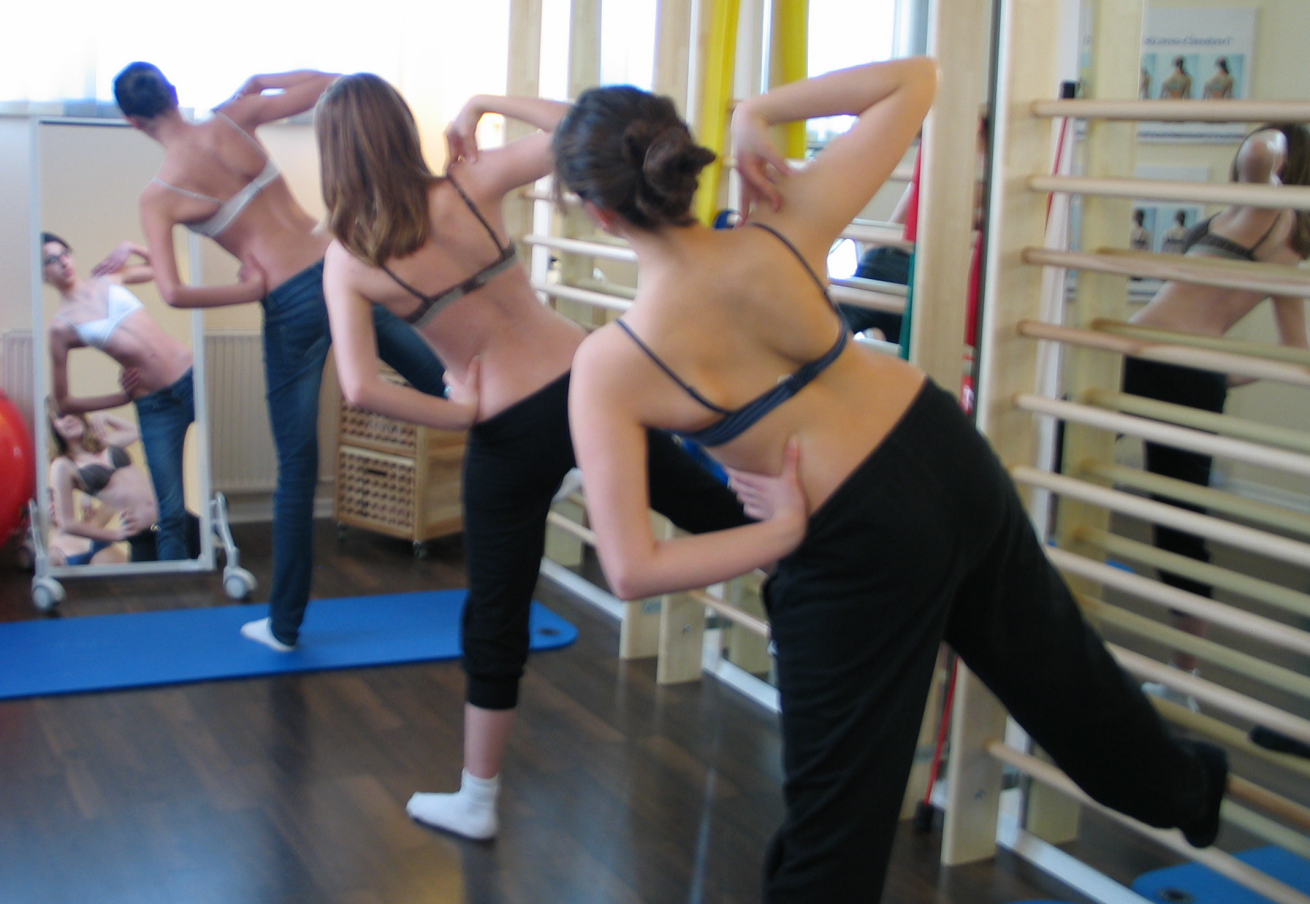
**Starting position of the ‚muscle cylinder' exercise**. Before starting the original exercise an automated postural correction is achieved in the asymmetric starting position by using the corrective postural reflex activation [12].

In horizontal starting positions these precorrections due to postural reflex activity could not be achieved and therefore these postural corrections cannot be regarded as effective in beginning an exercise in asymmetric upright postition.

The programme was getting more complex and complicated during the 90's, but a clear direction of development was no longer visible. While brace treatment constantly developed and improved, the exercise programme lost its effectiveness compared to other centres after the Katharina Schroth Klinik was taken over by Asklepios in 1995. The groups of sometimes 15-16 patients were too big for significant gains and with only one therapist.

The same programme was also performed at that time in the Elena Salva Institute in Barcelona under the supervision of Dr. Manuel Rigo. Together with the author he improved many parts of the original programme according to the latest knowledge throughout the 90's.

He also offered more intensive courses with groups of 10 patients and two or three therapists at the same time and was able to achieve significant postural improvements also exceeding the margins of technical error measured with the help of the Formetric system [26,27]. While Dr. Rigo's patients only received half of the treatment time than those patients in the Asklepios centre, they clearly had better outcomes with a similar program compared to the results published 1999 [28] not exceeding the technical error [29].

#### Courses for therapists

At the end of the 80's the author began a training programme for professionals and soon Dr. Rigo was one of the most important international instructors. He brought the original progamme to the US and the UK, thus distributing the knowledge worldwide together with the author, investigating the outcome of such treatment [10,11,17-25]. Consequently, the Schroth programme is now known and recognized all over the world.

#### Recent developments

Content, rehabilitation times and patients meanwhile have changed, while braces today have been developed to offer highest treatment securitiy [30].

Therefore, today bracing in the patient at risk has to be regarded as the primary treatment (Figure 21 and 22). We have been able to cut the training times by adapting the old techniques [31] and introducing new forms of postural education (sagittal correction, ADL correction and experiencial learning) while the programme is still based on the original approaches of the 3-dimensional treatment according to Katharina Schroth, namely specific postural correction, correction of the scoliotic breathing patterns and correction of postural perception [32].

**Figure 21:**
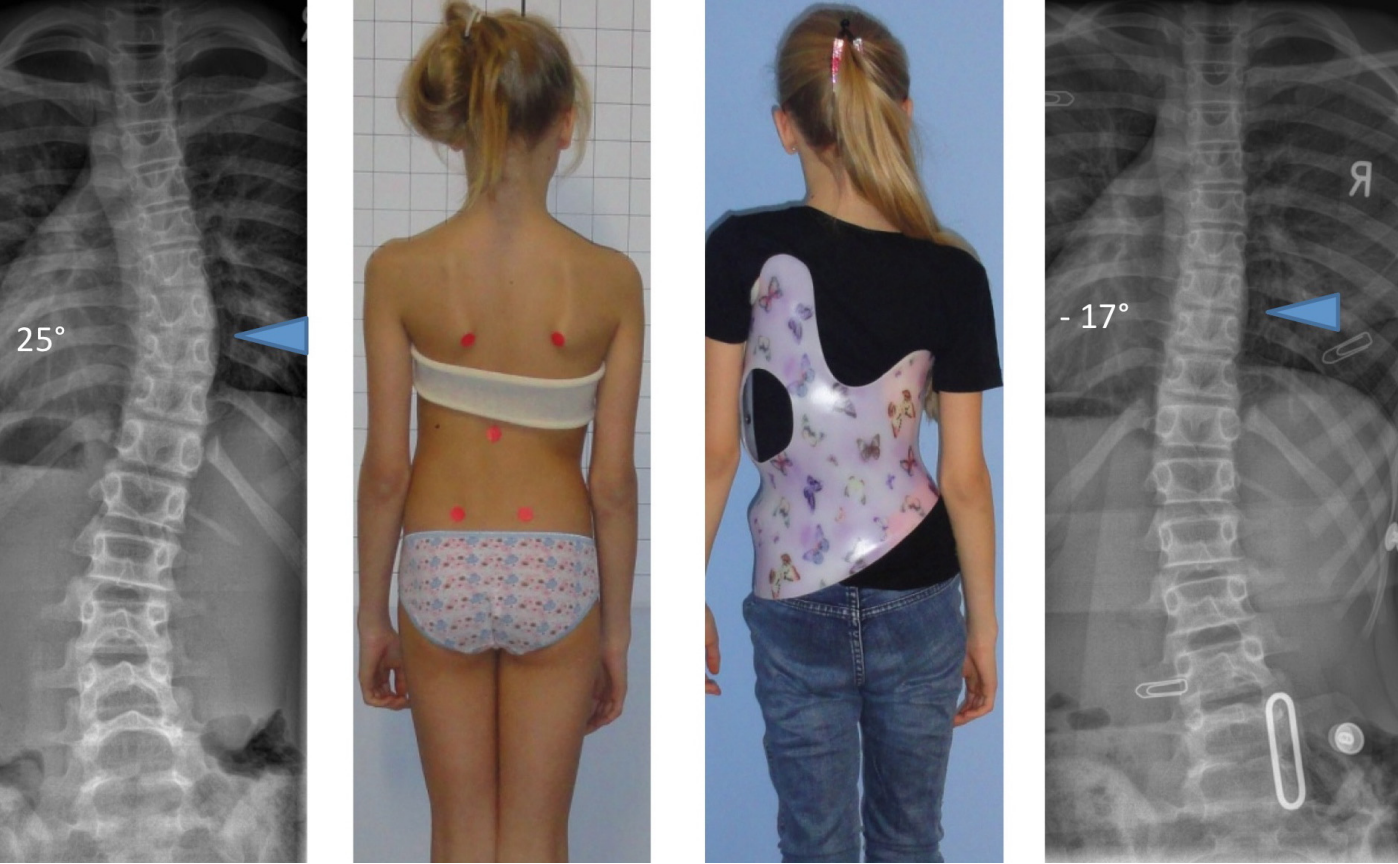
**Good in-brace correction in todays' bracing standard**. An overcorrection has been achieved in the single thoracic curve pattern.

**Figure 22:**
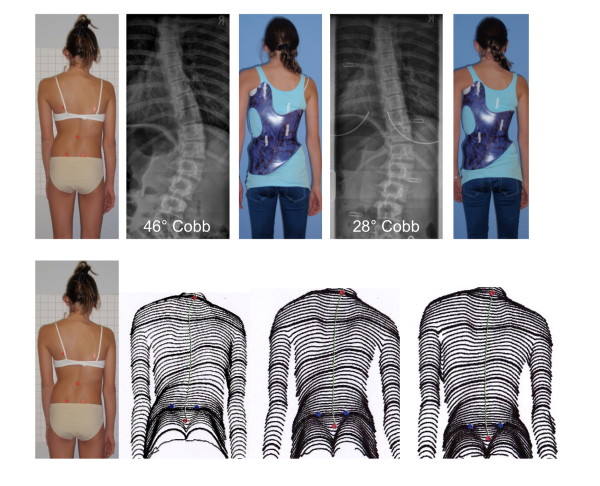
**Good in-brace correction in todays' bracing standard**. A sufficient correction has been achieved in the single thoracolumbar curve pattern. After 6 months of treatment the improvement of the trunk deformity is clearly visible in the surface topography scans (lower line of pictures).

Today, especially towards the end of the bracing period, more intensive physiotherapy is advisable. However todays' programme focusses on the activities of daily living (ADL) in order to avoid losing postural control in everday activity.

30 minutes of exercising is useless when the curve is loaded during the rest of the day.

Unloading the spine and curve therefore, is the major aim of the new programme derived from the original. The exercises today are regarded to be important for gaining postural control but not as an exercise per se.

Nevertheless, the programme has been improved with respect to the correction of the sagittal plane and today we are again fostering a postural correction to the highest possible degree (Figure 23) and here the circle closes again when we look at the old pictures with remarkable corrections achieved in really large curvatures (Figure 24 and 25).

**Figure 23:**
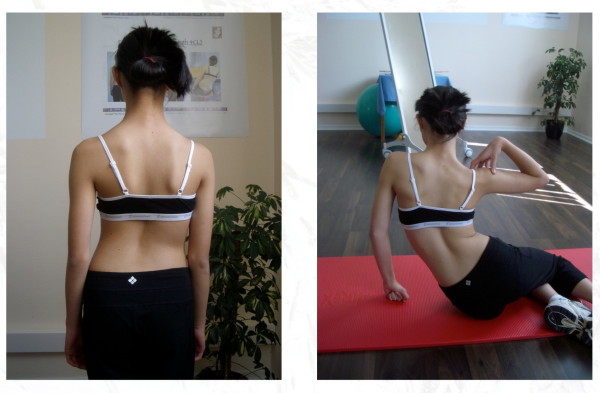
**Clinical overcorrection of a patients with a thoracic curve exceeding 40° in the ‚New Power Schroth' exercise called ‚Frog at the pond' **[12].

**Figure 24:**
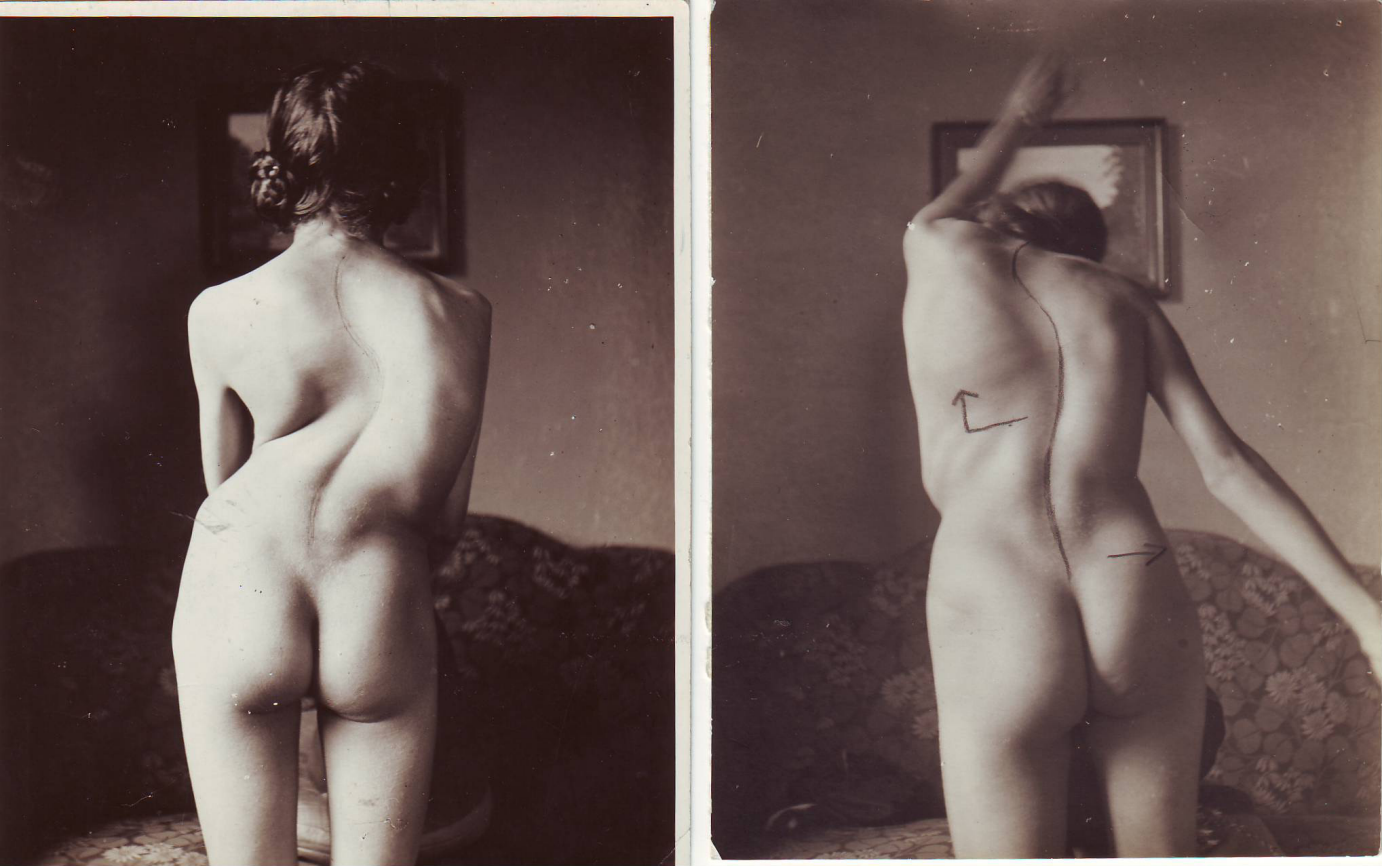
**Good correction effect during an original Schroth exercise in a patient with a very large rib hump**. This was a corrective exercise during the initial development of the original Schroth programme. Later on the exercises were performed differently. [Historical picture from the picture database of Christa Lehnert-Schroth, Meissen 20's].

**Figure 25:**
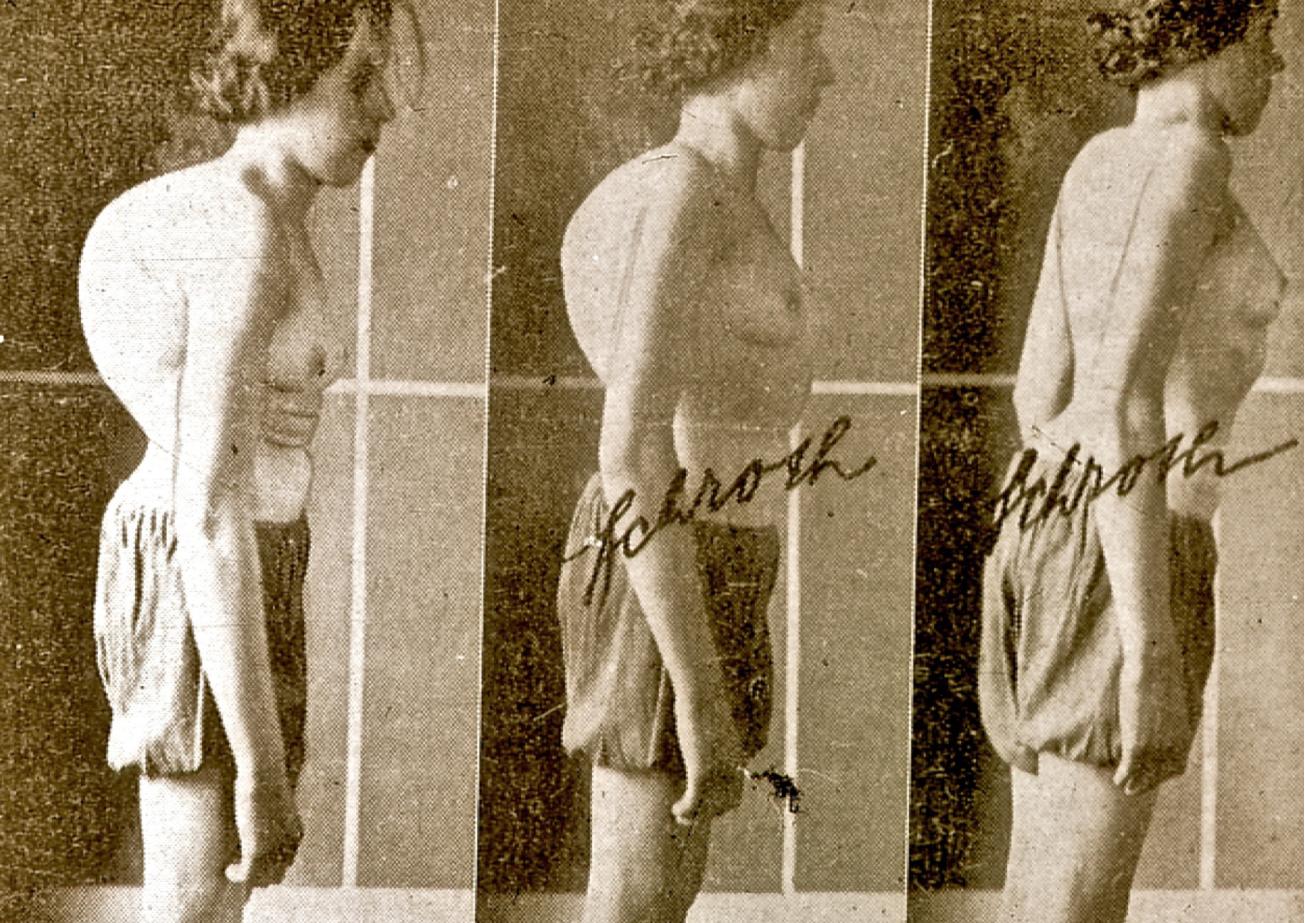
**Impressive correction of a significant rib hump during an intensive rehabilitation of 3-months in the old institute in Meissen, East Germany before WW II**. The picture on the right is in actice correction. [Historical picture from the picture database of Christa Lehnert-Schroth, Meissen 20's].

Todays ‚New Power Schroth' programme is designed for small and moderate curvatures. Once a thoracic curve exceeds 70° of course the original Schroth programme seems to offer the greatest advantage for the patient.

In conclusion: The original concept of Katharina Schroth was, and still is, the appropriate programme to address large curvatures, especially main thoracic curves.

The latest developments (‚New Power Schroth' as part of the Scoliologic™'Best Practice' programme) are designed for small and moderate curves-nowadays the main indication for physiotherapy. For this new programme, however rehabilitation times of more than one week are no longer necessary [30,33].

The basic principles of the original Schroth concept are still in use today, though adapted to latest evidence. Also the original Lehnert Schroth classification (Figure 3) is still in use today but it was augemented to meet the needs arising while applying pattern specific braces of the lates standard most precisely (Figure 26).

**Figure 26:**
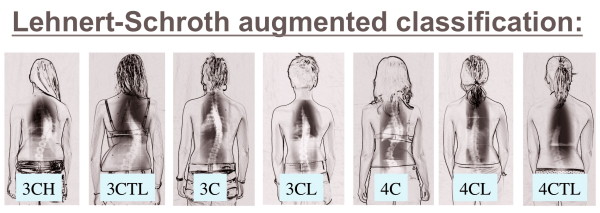
**The new augmented classification according to Lehnert-Schroth**. This classification is still based on the classical one (Figure. 3), however more precise. Today this is a big help in certain cases during physiotherapy, but also the basic classification in the application of the Gensingen brace™ [12].

## Competing interests

The author is advisor of Koob-Scolitech, Abtweiler, Germany.

## Supplementary Material

Additional file 1 **Description of the steps to scoliosis correction and also the description of contraindications as well**. (Original manuscript by Katharina Schroth in German). This file is not translated and used for documentation only [Historical picture from the picture database of Christa Lehnert-Schroth]. Click here for file

Additional file 2 **Description of the steps to scoliosis correction and also the description of contraindications as well**. (Original manuscript by Katharina Schroth in German). This file is not translated and used for documentation only [Historical picture from the picture database of Christa Lehnert-Schroth]. Click here for file
