# Supplementary figures and images for: The method of Katharina Schroth - history, principles and current development
Source: Scoliosis. 2011 Aug 30;6:17. doi: 10.1186/1748-7161-6-17 (PMC3180431; doi:10.1186/1748-7161-6-17)

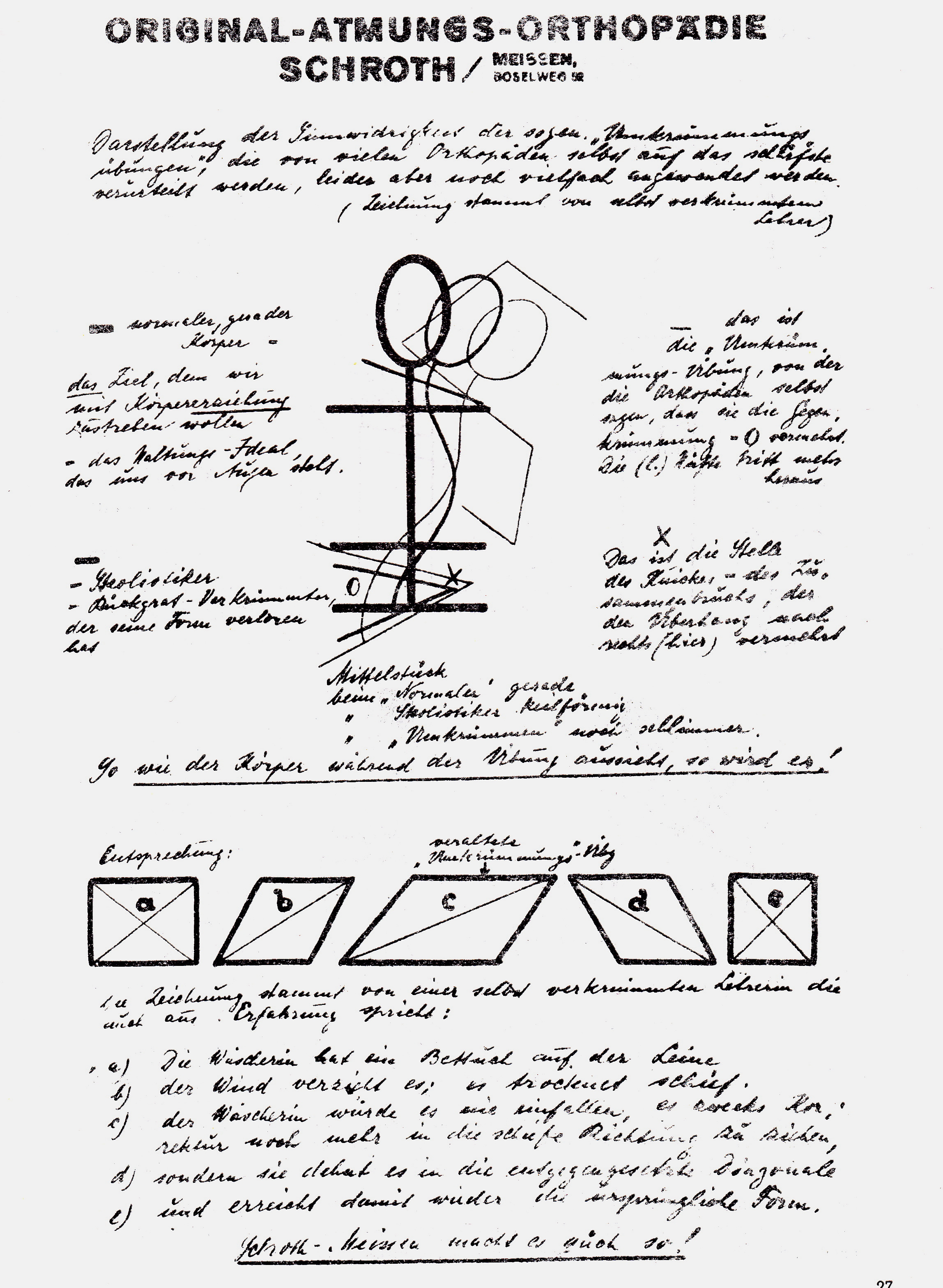

Supplement: Additional file 1 — Description of the steps to scoliosis correction and also the description of contraindications as well. (Original manuscript by Katharina Schroth in German). This file is not translated and used for documentation only [Historical picture from the picture database of Christa Lehnert-Schroth]. [file 1748-7161-6-17-S1.JPEG]

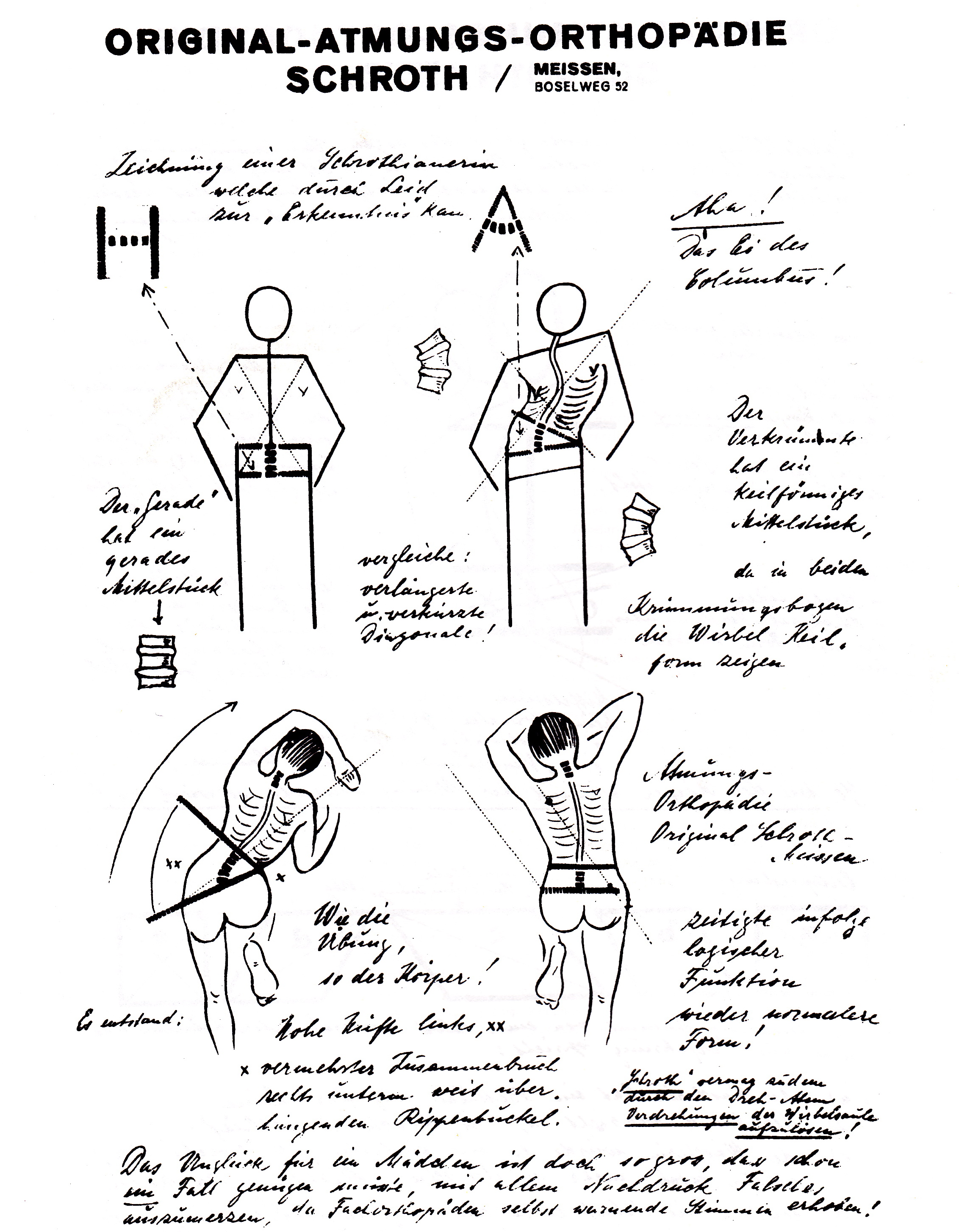

Supplement: Additional file 2 — Description of the steps to scoliosis correction and also the description of contraindications as well. (Original manuscript by Katharina Schroth in German). This file is not translated and used for documentation only [Historical picture from the picture database of Christa Lehnert-Schroth]. [file 1748-7161-6-17-S2.JPEG]
